# Color Image Restoration Using Sub-Image Based Low-Rank Tensor Completion

**DOI:** 10.3390/s23031706

**Published:** 2023-02-03

**Authors:** Xiaohua Liu, Guijin Tang

**Affiliations:** 1 College of Electronic and Optical Engineering & College of Flexible Electronics (Future Technology), Nanjing University of Posts and Telecommunications, Nanjing 210023, China; 2 Jiangsu Key Laboratory of Image Processing and Image Communication, Nanjing University of Posts and Telecommunications, Nanjing 210003, China

**Keywords:** sub-image, low rank, tensor completion, image restoration

## Abstract

Many restoration methods use the low-rank constraint of high-dimensional image signals to recover corrupted images. These signals are usually represented by tensors, which can maintain their inherent relevance. The image of this simple tensor presentation has a certain low-rank property, but does not have a strong low-rank property. In order to enhance the low-rank property, we propose a novel method called sub-image based low-rank tensor completion (SLRTC) for image restoration. We first sample a color image to obtain sub-images, and adopt these sub-images instead of the original single image to form a tensor. Then we conduct the mode permutation on this tensor. Next, we exploit the tensor nuclear norm defined based on the tensor-singular value decomposition (t-SVD) to build the low-rank completion model. Finally, we perform the tensor-singular value thresholding (t-SVT) based the standard alternating direction method of multipliers (ADMM) algorithm to solve the aforementioned model. Experimental results have shown that compared with the state-of-the-art tensor completion techniques, the proposed method can provide superior results in terms of objective and subjective assessment.

## 1. Introduction

In recent years, image restoration methods using low-rank models have achieved great success. However, how do we construct a low-rank tensor? In image restoration, the most common method is to use the nonlocal self-similarity (NSS) of images. It uses the similarity between image patches to infer missing signal components. Similar patches are collected into a group so that these blocks in each group can have a similar structure to approximately form a low-rank matrix/tensor, and the image is restored by exploiting a low-rank prior in the matrix/tensor composed of similar patches [1,2]. However, when an image lacks enough similar components, or its similar components are damaged by noise, the quality of the reconstructed image will be poor. Therefore, in some cases, the method of using NSS to find similar blocks to construct low-rank tensors is not feasible. In addition, the large-scale searching of NSS patches is very time-consuming, which will affect the efficiency of the reconstruction algorithm.

It is well known that most high-dimensional data such as color images, videos, and hyperspectral images, can naturally be represented as tensors. For example, a color image with a resolution of 512-by-512 can be represented as a 512-by-512-by-3 tensor. Because of the similarity of tensor content, it is considered to be low-rank [3]. Especially, many images that contain many texture regions are often low rank. Nowadays, in most low-rank tensor completion (LRTC) algorithms, the low-rank constraint is performed on the whole of the high-dimensional data, not a part of it. Many algorithms follow this idea. These typical algorithms include fast low rank tensor completion (FaLRTC) [4], LRTC based tensor nuclear norm (LRTC-TNN) [5], the low-rank tensor factorization method (LRTF) [6], and the method of integrating total variation (TV) as regularization term into low-rank tensor completion based on tensor-train rank-1 (LRTV-TTr1) [7]. However, this simple representation does not make full use of the low-rank nature of this data. In this paper, we propose a novel method called sub-image based low-rank tensor completion (SLRTC) for image restoration. To start with, we utilize the local similarity in sampling an image to obtain a sub-image set which has a strong low-rank property. We use this sub-image set to recover the low-rank tensor from the corrupted observation image. In addition, the tensor nuclear norm is direction-dependent: the value of the tensor nuclear norm may be different if a tensor is rotated or its mode is permuted. In our completion method, the mode (row × column × RGB) of a third-order tensor is permuted to the mode with RGB in the middle (row × RGB × column), and then the low-rank optimization completion is performed on the permuted tensor. Finally, the alternating direction method of multipliers is used to solve the problem.

The main contributions of this paper can be summarized as follows:

- We propose a novel framework of sub-image based low-rank tensor completion for color image restoration. The tensor nuclear norm is based on the tensor tubal rank (TTR), which is obtained by the tensor-singular value decomposition (t-SVD) in this framework. In order to achieve the stronger low-rank tensor, we sample each channel of a color image into four sub-images, and use these sub-images instead of the original single image to form a tensor.
- The optimization completion is performed on the permuted tensor in the proposed framework. The mode of a third-order tensor of a color image is usually denoted by (row × column × RGB). It is permuted to the mode (row × RGB × column) in our framework. This permutation operation can make a better restoration and decrease the running time.

The remainder of the paper is organized as follows. Section 2 introduces the definitions and gives the basic knowledge about the t-SVD decomposition. In Section 3, we propose a novel model of low-rank tensor completion, and use the standard alternating direction method of multipliers (ADMM) algorithm to solve the model. In Section 4, we compare our model with other algorithms, and analyse the performance of the proposed method. Finally, we draw the conclusion of our work in Section 5.

## 2. Related Work and Foundation

This section mainly introduces some operator symbols, related definitions, and theorems of the tensor SVD.

### 2.1. Notations and Definitions

For convenience, we first introduce the notations that will be extensively used in the paper. $X\in {\mathbb{R}}^{I\times J\times K}$ (each element can be written as $X_{ijk}$ or X(i,j,k)) represents a third-order tensor, and the real number field and the complex number field are represented as ℝ and ℂ. And X(i,:,:),X(:,j,:) and X(:,:,k) are the horizontal slice, lateral slice and frontal slice of the third-order tensor, respectively. For simplicity, we denote the *k*-th frontal slice X(:,:,k) as $X^{\left(k\right)}$. For $X\in {\mathbb{R}}^{I\times J\times K}$, we denote $\hat{X}\in {\mathbb{C}}^{I\times J\times K}$ as the discrete Fourier transform (DFT) results of all tubes of X. By using the Matlab function *fft*, we get $\hat{X}=fft(A,[],3)$. Similarly, we denote the *k*-th frontal slice $\hat{X}(:,:,k)$ as ${\hat{X}}^{\left(k\right)}$.

### 2.2. Tensor Singular Value Decomposition

Recently, the tensor nuclear norm that is defined based on tensor singular value decomposition (t-SVD) has shown that it can effectively utilize the inherent low-rank structure of tensors [8,9,10]. Let $M\in {\mathbb{R}}^{N_{1}\times N_{2}\times N_{3}}$ be an unknown low rank tensor, the entries of M are observed independently with probability p and Ω represents the set of indicators of the observed entries (i.e., if (i,j,k)∈Ω, X(i,j,k)=M(i,j,k); else X(i,j,k)=0). So, the problem of tensor completion is to recover the underlying low rank tensor M from the observations {$M_{ijk},(i,j,k)\in \Omega$}, and the corresponding low-rank tensor completion model can be written as:

(1) $$\arg\underset{X}{\min}{\left\|X\right\|}_{*},s.t.\;P_{\Omega }(X)=P_{\Omega }(M),$$

where ${\left\|X\right\|}_{*}$ is the tensor nuclear norm (TNN) of tensor $X\in {\mathbb{R}}^{N_{1}\times N_{2}\times N_{3}}$.

The TNN-based model shows its effectiveness in maintaining the internal structure of tensors [11,12]. In many low-order tensor restoration tasks, low-tubal-rank models have achieved better performance than low-Tucker-rank models, such as tensor completion [13,14,15], tensor denoising [16,17], tensor robust principal component analysis [11,18], etc.

In order to enhance the low-rank feature of an image, we utilize the local similarity to sub-sample an image to obtain a sub-image set which has a strong low-rank property, and propose a sub-image based TNN model to recover low-rank tensor signals from corrupted observation images.

**Definition** **1** **(block** **circulant** **matrix** **[8]).** *For* $X\in {\mathbb{R}}^{I\times J\times K}$ *, the block circulant matrix* $bcirc(X)\in {\mathbb{R}}^{IK\times JK}$ *is defined as*

(2) $$bcirc(X)=\left[\begin{array}{llll} X^{\left(1\right)} & X^{\left(K\right)} & \cdots & X^{\left(2\right)}\\ X^{\left(2\right)} & X^{\left(1\right)} & \cdots & X^{\left(3\right)}\\ \vdots & \vdots & \ddots & \vdots \\ X^{\left(K\right)} & X^{\left(K-1\right)} & \cdots & X^{\left(1\right)} \end{array}\right]$$

**Definition** **2** **(unfold,** **fold** **[9]).** *For*$X\in {\mathbb{R}}^{I\times J\times K}$*, the tensor unfold and matrix fold operators are defined as*

(3) $$unfold(X)=\left[\begin{array}{l} X^{\left(1\right)}\\ X^{\left(2\right)}\\ \vdots \\ X^{\left(K\right)}\; \end{array}\right],fold(unfold(X))=X$$

where the *unfold* operation maps X to a matrix of size IK×J, and *fold* is its inverse operator.

**Definition** **3** **(T-product** **[8]).** *Let*$X\in {\mathbb{R}}^{N_{1}\times N_{2}\times N_{3}}$*and*$Y\in {\mathbb{R}}^{N_{2}\times t\times N_{3}}$*, then the T-product*$Z=X*Y\in {\mathbb{R}}^{N_{1}\times t\times N_{3}}$*is defined as*

(4) Z=fold(bcirc(X)⋅unfold(Y))

and

(5) $$Z\left(i,j,:\right)=\sum_{k=1}^{N_{2}}X\left(i,k,:\right)•Y\left(k,j,:\right)$$

where the operation • is circular convolution.

**Definition** **4** **(f-diagonal** **tensor** **[8]).** *If each frontal slice of a tensor is a diagonal matrix, it is called f-diagonal tensor.*

**Definition** **5** **(t-SVD** **[8]).** *Let*$X\in {\mathbb{R}}^{N_{1}\times N_{2}\times N_{3}}$*, then it can be factored as*

(6) $$X=U*S*V^{*}$$

where $U\in {\mathbb{R}}^{N_{1}\times N_{1}\times N_{3}}$ and $V\in {\mathbb{R}}^{N_{2}\times N_{2}\times N_{3}}$ are orthogonal, and $S\in {\mathbb{R}}^{N_{1}\times N_{2}\times N_{3}}$ is an f-diagonal tensor.

The frontal slice of $\hat{X}$ has the following properties:

(7) $$conj({\hat{X}}^{\left(k\right)})={\hat{X}}^{\left(N_{3}-k+2\right)},\;k=2,\cdots ,\left\lfloor \frac{N_{3}+1}{2}\right\rfloor$$

where $\left\lfloor \cdot \right\rfloor$ represents the downward integer operator.

We can effectively obtain the t-SVD by calculating a series of matrix SVDs in the Fourier domain.

**Definition** **6** **(tensor** **tubal** **rank** **[16]).** *For* $X\in {\mathbb{R}}^{N_{1}\times N_{2}\times N_{3}}$ *, the tensor tubal rank is denoted as* $rank_{t}(X)$ *, which is defined as the number of non-zero singular tubes of* S *, where* S *is the t-SVD decomposition of* X *, namely*

(8) $$rank_{t}(X)=\#\{i,S(i,i,:)\neq 0\}$$

**Definition** 7 **(tensor** **average** **rank [18]).** *For* $X\in {\mathbb{R}}^{N_{1}\times N_{2}\times N_{3}}$ *, the tensor tubal rank is denoted as* $rank_{a}(X)$ *, is defined as*

(9) $$rank_{a}(X)=\frac{1}{N_{3}}rank(\mathrm{bcirc}(X))=\frac{1}{N_{3}}{\left\|X\right\|}_{*}$$

**Definition** **8** **(tensor** **nuclear** **norm** **[18]).** *For*$X\in {\mathbb{R}}^{N_{1}\times N_{2}\times N_{3}}$*, the tensor nuclear norm of*A*is defined as*

(10) $${\left\|X\right\|}_{*}=\sum_{i=1}^{r}S(i,i,1)$$

where $r=rank_{t}(X)$.

## 3. Proposed Model

In this section, we propose a sub-image tensor completion framework based on the tensor tubal rank for image restoration.

### 3.1. Sub-Image Generation

**Figure 1:**
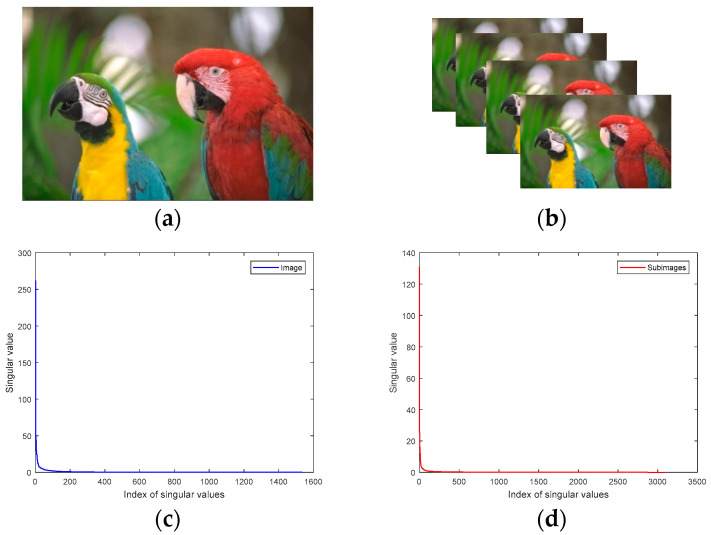
Color image and its singular values. (**a**) Color Image *kodim23* denoted by $X\in {\mathbb{R}}^{512\times 768\times 3}$; (**b**) Color sub-image set denoted by $X_{s}\in {\mathbb{R}}^{256\times 384\times 12}$; (**c**) the singular values of bcirc(X); (**d**) the singular values of $\mathrm{bcirc}(X_{s})$.

As we all know, real color images can be approximated by low-rank matrices on the three channels independently. If we regard a color image as a third-order tensor, and each channel corresponds to a frontal slice, then it can be well approximated by a low-tubal-rank tensor. Figure 1 shows an example to illustrate that most of the singular values of the corresponding tensor of an image are zero, so a low-tubal-rank tensor can be used to approximate a color image.

**Figure 2:**
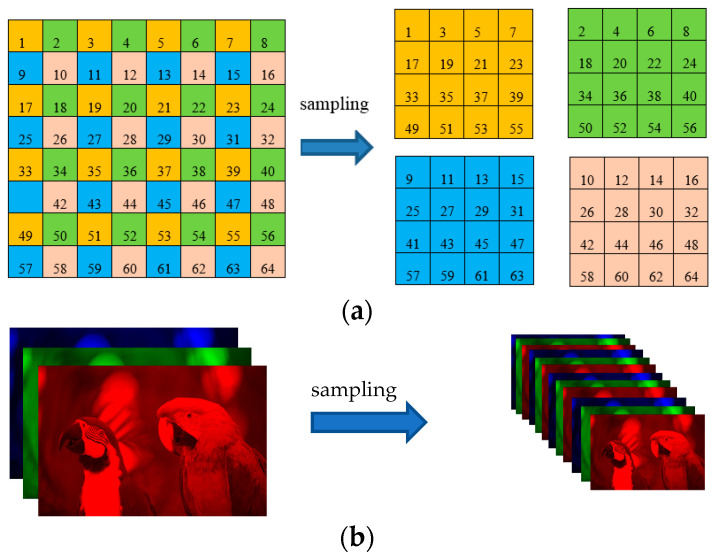
A simple demonstration of the sampling method. (**a**) An image is sampled to obtain four sub-images; (**b**) A three-channel RGB image is sampled to form four three-channel sub-images.

Although the aforesaid representation can approximate a color image, it does not make full use of the similarity of image data. In order to enhance its low-rank property, we sampled an image to obtain four similar images (All sampling factors in this paper are horizontal sampling factor: vertical sampling factor = 2:2), and each image is divided into four sub-images, and there is no pixel overlap between the sub-images, as shown in Figure 2a. Each small square represents a pixel. For a three-channel RGB image, its sampling method is illustrated in Figure 2b.

According to the prior knowledge of image local similarity, the four sub-images are similar, so they are composed of a sub-image tensor which has a low-rank structure. It should be noted that if the pixels of the image rows and columns are not even, we can add one row or one column and then do the down-sampling processing. It can be seen that the tensor representation of the color image kodim23 is $A\in {\mathbb{R}}^{512\times 768\times 3}$ in Figure 1. After sampling, we get the sub-image set denoted by $A_{s}\in {\mathbb{R}}^{256\times 384\times 12}$. Here we give the singular values of the tensor $\mathrm{bcirc}(A_{s})$ as shown in Figure 1d. Compared with Figure 1c, it can be seen that most of the singular values of the corresponding tensor of the sub-image set appear to be smaller. Therefore, compared with the original whole image, the sub-image data has stronger property of low rank.

### 3.2. Mode Permutation

It is important to note that the TNN is orientation-dependent. If the tensor rotates, the value of TNN and the tensor completion results from Formula (1) may be quite different. For example, a three-channel color image of size $n_{1}\times n_{2}$ can be represented as three types of tensors, i.e., $X_{1}\in {\mathbb{R}}^{n_{1}\times n_{2}\times 3}$,$X_{2}\in {\mathbb{R}}^{n_{1}\times 3\times n_{2}}$ and $X_{2}{}^{*}\in {\mathbb{R}}^{3\times n_{1}\times n_{2}}$, where $X_{1}$ is the most common image tensor representation, $X_{2}{}^{*}$ denotes the conjugate transpose of $X_{2}$, and ${\left\|X_{1}\right\|}_{*}\neq {\left\|X_{2}\right\|}_{*}$, ${\left\|X_{2}\right\|}_{*}={\left\|X_{2}{}^{*}\right\|}_{*}$.

**Figure 3:**
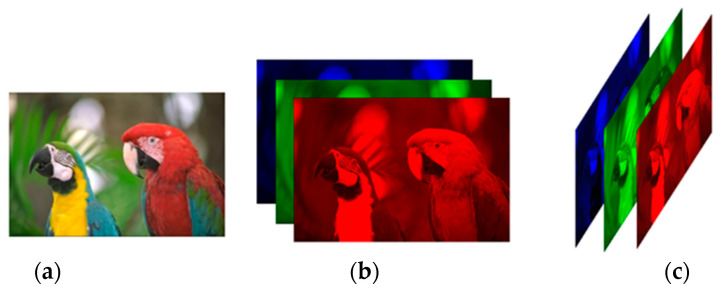
Two tensor representations of a color image. (**a**) image kodim23; (**b**) $X_{1}\in {\mathbb{R}}^{n_{1}\times n_{2}\times 3}$; (**c**) $X_{2}\in {\mathbb{R}}^{n_{1}\times 3\times n_{2}}$.

In order to further improve the performance, we perform the mode permutation [19] after sampling. Here we give an example of the mode permutation as shown in Figure 3.

In Figure 3, the size of the color image *kodim23* is $n_{1}\times n_{2}$. Its tensor representation is $X_{1}\in {\mathbb{R}}^{n_{1}\times n_{2}\times 3}$. After the mode permutation, it is denoted by $X_{2}\in {\mathbb{R}}^{n_{1}\times 3\times n_{2}}$. So $X_{2}\in {\mathbb{R}}^{n_{1}\times 3\times n_{2}}$ is called the mode permutation of $X_{1}\in {\mathbb{R}}^{n_{1}\times n_{2}\times 3}$.

The mode permutation option can avoid scanning an entire image, which reduces the overall computational complexity [19].

### 3.3. Solution to the Proposed Method

For the completion problem of the color image tensor $X\in {\mathbb{R}}^{N_{1}\times N_{2}\times N_{3}}$, we propose a color sub-image tensor $X_{s}\in {\mathbb{R}}^{n_{1}\times n_{2}\times n_{3}}$ (where $n_{1}=N_{1}/2$; $n_{2}=N_{2}/2$; $n_{3}=4N_{3}$) low-rank optimization model:

(11) $$\arg\underset{X_{s}}{\min}{\left\|X_{s}\right\|}_{*},s.t.\;P_{\Omega }(X_{s})=P_{\Omega }(M_{s})$$

where ${\left\|X_{s}\right\|}_{*}$ is the tensor nuclear norm of $X_{s}$, $M_{s}$ and $X_{s}$ are third-order tensors of the same size.

The problem (11) can be solved by the ADMM [20], where the key step is to calculate the proximity operator of the TNN, namely:

(12) $$prox_{\lambda {\left\|\;.\;\right\|}_{*}}(Y)=\arg\underset{X}{\min}\lambda {\left\|X\right\|}_{*}+\frac{1}{2}{\left\|X-Y\right\|}_{F}^{2}$$

According to the literature [18], let $Y=U*S*V^{*}$ be the t-SVD of $Y\in {\mathbb{R}}^{n_{1}\times n_{2}\times n_{3}}$, and for each λ>0, define the tensor singular value threshold (t-SVT) operator, as follows:

(13) $$D_{\lambda }(Y)=U*S_{\lambda }*V^{*}$$

where $S_{\lambda }=\mathrm{ifft}({\left(\hat{S}-\lambda \right)}_{+}\;,[],3)$, ${\left(\hat{S}-\lambda \right)}_{+}=\max((\hat{S}-\lambda ),0)$. It is worth noting that the t-SVT operator only needs to apply a soft threshold rule to the singular values $\hat{S}$ (instead of S) of the frontal slice of $\hat{Y}$. The t-SVT operator is a proximity operator related to TNN.

**Figure 4:**
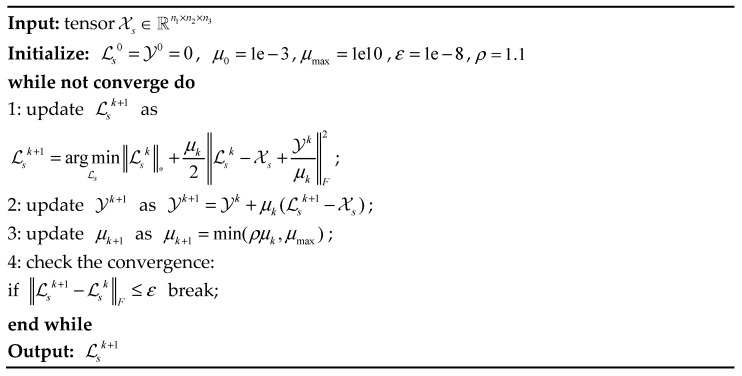
Our algorithm SLRTC.

Based on t-SVT, we exploit the ADMM algorithm to solve the problem of (11). The augmented Lagrangian function of (11) is defined as

(14) $$L(L_{s},Y,\mu )={\left\|L_{s}\right\|}_{*}+<Y,L_{s}-X_{s}>+\frac{\mu }{2}{\left\|L_{s}-X_{s}\right\|}_{F}^{2}$$

where $Y\in {\mathbb{R}}^{n_{1}\times n_{2}\times n_{3}}$ is the Lagrangian multiplier and μ>0 is the penalty parameter. We then update $L_{s}$ by alternately minimizing the augmented Lagrangian function L. The sub-problem has a closed-form solution, with the t-SVT operator related to TNN. A pseudo-code description of the entire optimization problem (11) is given in Figure 4.

In the whole procedure of algorithm SLRTC, the main per-iteration cost lies in the update $L_{s}{}^{k+1}$, which requires computing fast Fourier transform (FFT) and $\left\lceil \frac{n_{3}+1}{2}\right\rceil$ SVDS of $n_{1}\times n_{2}$ matrices. The per-iteration complexity is $O(n_{1}n_{2}n_{3}\log n_{3}+n_{\left(1\right)}n_{\left(2\right)}^{2}n_{3})$.

**Figure 5:**
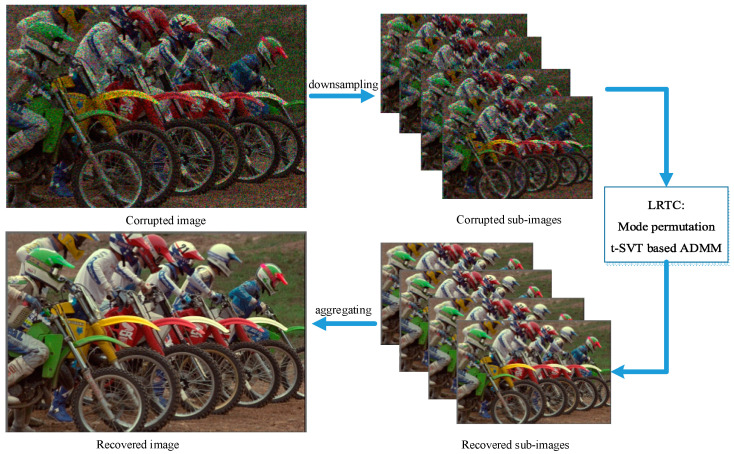
Flowchart of the proposed framework. The downsampling method is as shown in Section 3.1. After the mode permutation of the sub-images, t-SVT is performed to obtain the recovered sub-images. Finally, the final recovered image can be obtained by aggregating the recovered sub-images.

Therefore, the overall framework process of color image restoration based on sub-image low-rank tensor completion proposed is shown in Figure 5.

## 4. Experiments

In this section, we will compare the proposed SLRTC with several classic color image restoration methods (including FaLRTC [4], LRTC-TNN [5], TCTF [6] and LRTV-TTr1 [7]). Among them, the frontal slice of the input tensor in the LRTC-TNN1 method corresponds to R, G, and B channels, while the lateral slice of the input tensor in the LRTC-TNN2 method corresponds to R, G, and B channels; the LRTC-TNN method is based on TNN, which solves the tensor completion problem by solving the convex optimization.

To evaluate the performance of different methods for color image restoration, we used the widely used peak signal-to-noise ratio (PSNR) and structure similarity (SSIM) [21] indicators in this experiment.

### 4.1. Color Image Recovery

**Figure 6:**
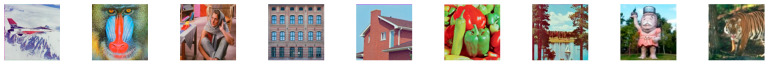
Ground truth of nine benchmark color images: Airplane, Baboon, Barbara, Façade, House, Peppers, Sailboat, Giant and Tiger (from left to right).

We first use the original real nine color images for testing, as shown in Figure 6.

The size of each image is 256 × 256 × 3, i.e., row × column × RGB. In order to test the repair effect of various algorithms on the images, we randomly lost 30%, 50%, and 70% of the pixels in each image, and formed an incomplete tensor $X\in {\mathbb{R}}^{256\times 256\times 3}$.

Table 1 lists the PSNR and SSIM comparisons of all methods to restore the images. Compared with the LRTC-TNN1, LRTC-TNN2 and TCTF methods, the SLRTC method can usually obtain the best image restoration results when the missing rate is 30%, 50%, and 70%. By analysing the specific data in Table 1, it can be seen that when the local smoothing in the image accounts for a large proportion, such as Airplane, Pepper and Sailboat, the effect will be better if the LRTV-TTr1 method is utilized to restore the images, because the advantage of TV regularization is to make use of the local smoothness of the image. In addition, when the missing rate is higher (greater than or equal to 70%), the LRTV-TTr1 method is better than SLRTC. When the image contains a large number of texture regions, that is, the image itself has a strong low rank, the best effect can be achieved by applying low rank constraints to the restoration of degraded images, such as *facade*.

In order to further verify the effectiveness of the proposed algorithm, we additionally used 24 color images from the Kodak PhotoCD dataset (http://r0k.us/graphics/kodak/ (accessed on 3 January 2018)) for testing. The size of each image is 768×512×3. As in the previous test, in order to test the repair effect of various algorithms on the image, we randomly lost 30%, 50%, and 70% of the pixels in each image, and formed an incomplete tensor $X\in {\mathbb{R}}^{768\times 512\times 3}$.

Table 2 lists the PSNR and SSIM comparisons of all methods to restore the images when the missing rate is 30%. The best values among all these methods are in boldface. It can be seen that our algorithm SLRTC can surpass the other algorithms in terms of PSNR, and the PSNR value is about 1.5-5db higher than the other methods. However, in terms of SSIM, it can be seen that the LRTV-TTr1 method is basically optimal.

Table 3 and Table 4 are the comparison of PSNR and SSIM when the missing rate is 50% and 70%, respectively. As shown in Table 2, our algorithm can surpass the other algorithms in terms of PSNR, but the SSIM value of image restoration is lower than that of the LRTC-TTr1 method. The reason is mainly due to the sampling process from image to sub-image in the first step of the SLRTC method. Although the sub-image has a stronger low rank than the original image, it weakens the overall structural relevance of the image.

**Figure 7:**
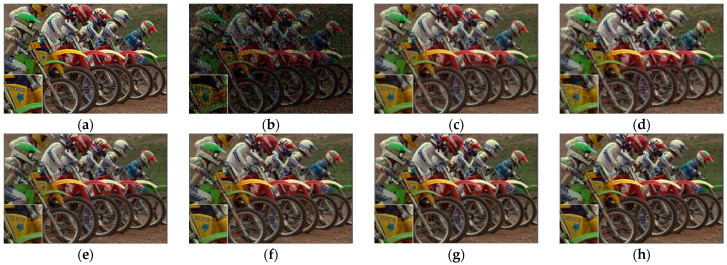
Visual effect comparison of different methods on the color image kodim05. The incomplete image contains 50% missing entries, shown as black pixels. (**a**) Original image; (**b**) Incomplete image; (**c**) FaLRTC (26.01 dB); (**d**) TCTF (22.45 dB); (**e**)LRTC-TNN1 (28.34 dB); (**f**) LRTC-TNN2 (29.98 dB); (**g**) LRTV-TTr1 (29.08 dB); (**h**) SLRTC (31.93 dB).

**Figure 8:**
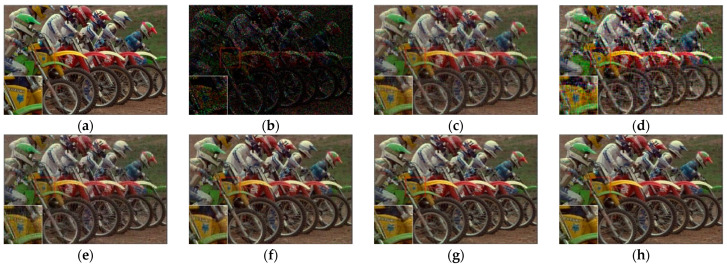
Visual effect comparison of different methods on the color image kodim05. The incomplete image contains 70% missing entries, shown as black pixels. (**a**) Original image; (**b**) Incomplete image; (**c**) FaLRTC (21.80 dB); (**d**) TCTF (19.41 dB); (**e**) LRTC-TNN1 (22.67 dB); (**f**) LRTC-TNN2 (24.59 dB); (**g**) LRTV-TTr1 (24.48 dB); (**h**) SLRTC (26.14 dB).

**Figure 9:**
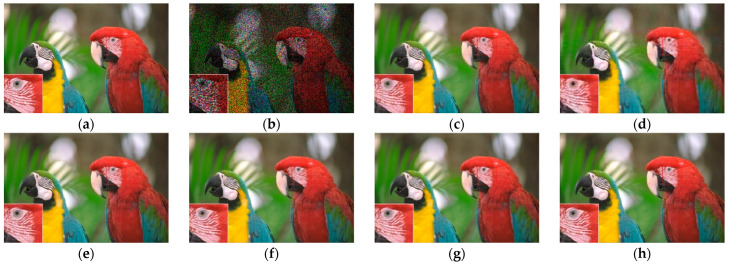
Visual effect comparison of different methods on the color image kodim23. The incomplete image contains 50% missing entries, shown as black pixels. (**a**) Original image; (**b**) Incomplete image; (**c**) FaLRTC (33.35 dB); (**d**) TCTF (29.21 dB); (**e**) LRTC-TNN1 (33.47dB); (**f**) LRTC-TNN2 (33.72 dB); (**g**) LRTV-TTr1 (34.46dB); (**h**) SLRTC (37.96dB).

**Figure 10:**
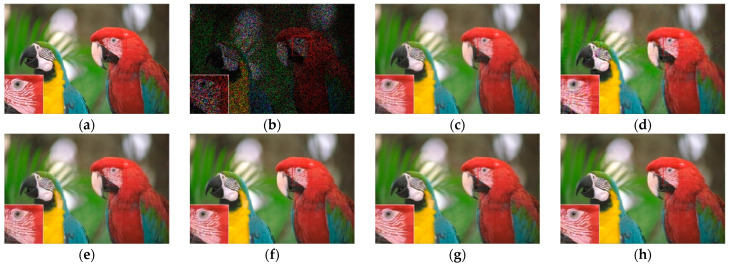
Visual effect comparison of different methods on the color image kodim23. The incomplete image contains 70% missing entries, shown as black pixels. (**a**) Original image; (**b**) Incomplete image; (**c**) FaLRTC (29.09 dB); (**d**) TCTF (26.94 dB); (**e**) LRTC-TNN1 (28.94 dB); (**f**) LRTC-TNN2 (29.14 dB); (**g**) LRTV-TTr1 (30.89 dB); (**h**) SLRTC (32.33 dB).

Figure 7, Figure 8, Figure 9 and Figure 10 show the comparison of visual quality when the missing rate is 50% and 70%. In contrast, the SLRTC method can better preserve the texture of the image.

**Figure 11:**
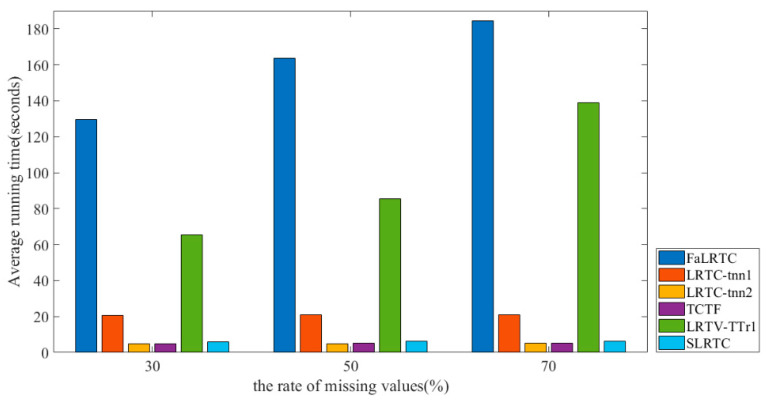
Comparison of the running time.

At the same time, we also give a comparison of the algorithm complexity of restoring 24 test images with a resolution of 768 × 512, as shown in Figure 11. It can be seen that SLRTC is much faster than the FaLRTC, LRTC-TNN1, and LRTV-TTr1 methods, and is comparable to the TCTF and LRTC-TNN2 methods.

### 4.2. Color Video Recovery

Next, we tested the performance of different methods in completing the task of video data. The test video sequences are City, Bus, Crew, Soccer, and Mobile. (https://engineering.purdue.edu/~reibman/ece634/ (accessed on 3 January 2018)).

The main consideration is the third-order tensor. Here, the following preprocessing is performed on each video: adjust the video size of 352×288×3×30 (row × column × RGB × number of frames) to a third-order tensor $X\in {\mathbb{R}}^{352\times 288\times 90}$.

**Figure 12:**
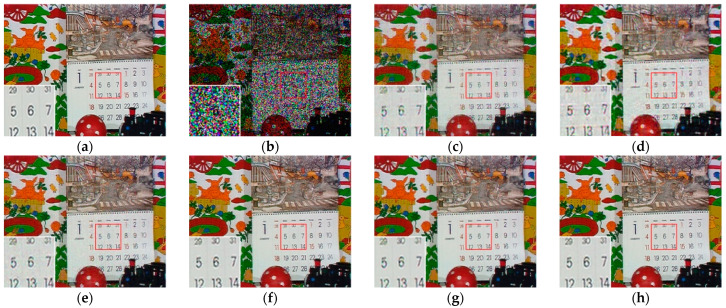
Visual effect comparison of different methods on the ninth frame completion of the Mobile video. The incomplete video contains 50% missing entries. (**a**) Original image; (**b**) Incomplete image; (**c**) FaLRTC (21.51 dB); (**d**) TCTF (21.80 dB); (**e**) LRTC-TNN1 (24.12 dB); (**f**) LRTC-TNN2 (28.09dB); (**g**) LRTV-TTr1 (26.15 dB); (**h**) SLRTC (29.97 dB).

**Figure 13:**
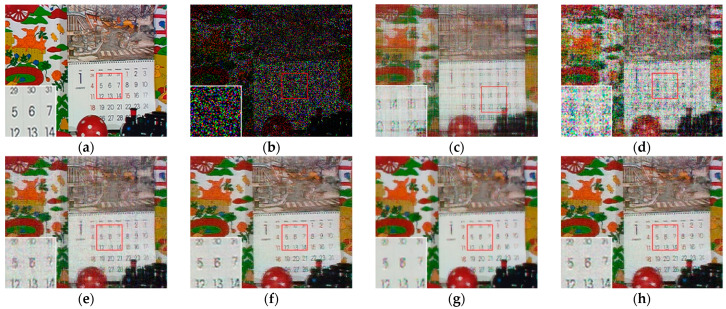
Visual effect comparison of different methods on the ninth frame completion of the Mobile video. The incomplete video contains 80% missing entries. (**a**) Original image; (**b**) Incomplete image; (**c**) FaLRTC (16.46 dB); (**d**) TCTF (11.53 dB); (**e**) LRTC-TNN1 (18.69 dB); (**f**) LRTC-TNN2 (20.67dB); (**g**) LRTV-TTr1 (19.91 dB); (**h**) SLRTC (20.87 dB).

**Figure 14:**
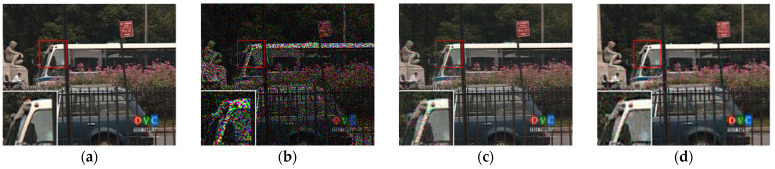

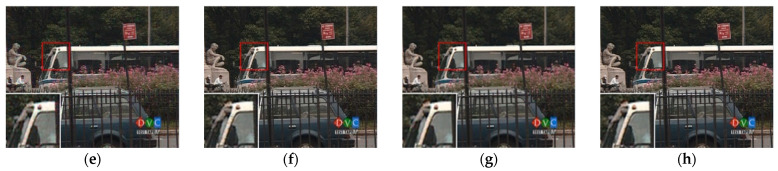
Visual effect comparison of different methods on the ninth frame completion of the Bus video. The incomplete video contains 50% missing entries. (**a**) Original image; (**b**) Incomplete image; (**c**) FaLRTC (27.30 dB); (**d**) TCTF (26.42 dB); (**e**) LRTC-TNN1 (29.05 dB); (**f**) LRTC-TNN2 (32.92 dB); (**g**) LRTV-TTr1 (31.77 dB); (**h**) SLRTC (34.34 dB).

**Figure 15:**
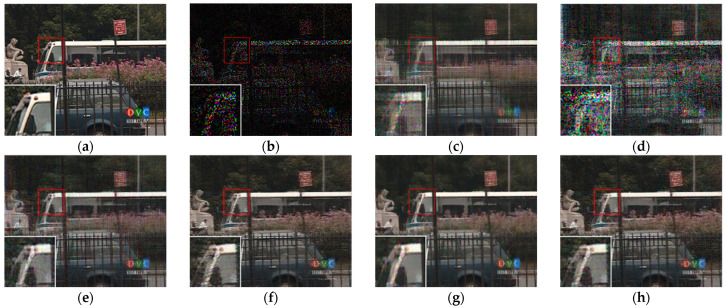
Visual effect comparison of different methods on the ninth frame completion of the Bus video. The incomplete video contains 80% missing entries. (**a**) Original image; (**b**) Incomplete image; (**c**) FaLRTC (21.42 dB); (**d**) TCTF (14.00 dB); (**e**) LRTC-TNN1 (22.51 dB); (**f**) LRTC-TNN2 (24.86 dB); (**g**) LRTV-TTr1 (23.99 dB); (**h**) SLRTC (24.69 dB).

Figure 12, Figure 13, Figure 14 and Figure 15 show the visual quality comparison of the *Mobile* and *Bus* video sequences repaired by different methods when the missing rate is 50% and 80%. When the missing rate is 50%, SLRTC can capture the inherent multi-dimensional characteristics of the data, and the video frame recovery effect is better than other methods; when the missing rate is 80%, the subjective visual quality of SLRTC in the *Mobile* video frame repair is better than other methods, while the subjective visual quality of the repair effect on the *Bus* video frame is not as good as LRTV_TTr1.

**Figure 16:**
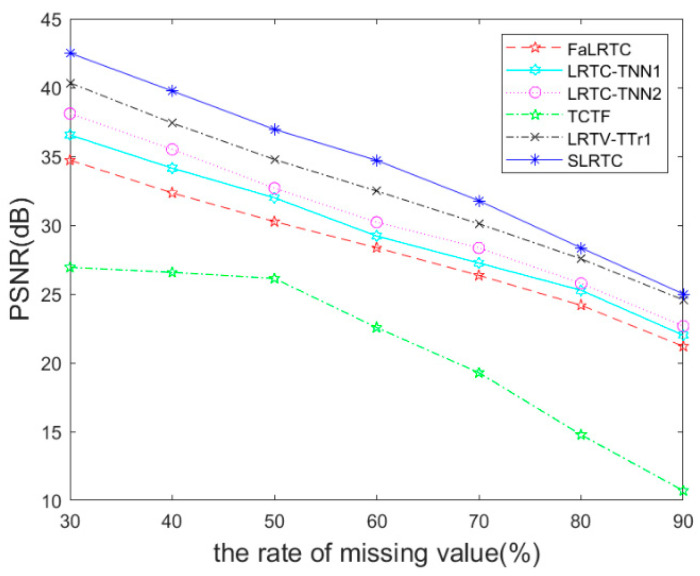
The PSNR metric on video data recovery.

Randomly removing 30%, 40%, 50%, 60%, 70%, 80%, and 90% pixels in the videos, Figure 16 shows the performance comparison of various methods for videos recovery. It can be seen that the SLRTC algorithm proposed is better than other methods.

## 5. Conclusions

This paper proposes a color image restoration method called SLRTC. Based on the nature of the tensor tubal rank, our method does not minimize the tensor nuclear norm on the observed image, but uses the local similarity characteristics of the image to decompose the image into multiple sub-images through downsampling to enhance the tensor of low rank. Experiments show that the proposed algorithm is better than the comparison algorithms in terms of color image restoration quality and running time.

Obviously, the method proposed in this paper is parameter-independent, and parameter adjustment usually requires complex calculations. In the absence of TV regularization terms, the method proposed in this paper uses the image partial smoothing prior to downsampling the image. It is well integrated into the sub-image low-rank tensor completion, and the effectiveness of the model is proved through experiments.

Since deep learning-based algorithms have shown their potential to tackle this problem of image restoration in recent years [22,23], we next will integrate the low-rank prior into the neural networks to achieve better performance.

## Figures and Tables

**Table 1:** The PSNR and SSIM Comparison of different methods on nine images (The best results are highlighted).

Test Images	Missngrate	FaLRTC [4]		LRTC-TNN1 [5]		LRTC-TNN2 [5]		TCTF [6]		LRTV-TTr1 [7]		SLRTC	
		PSNR	SSIM	PSNR	SSIM	PSNR	SSIM	PSNR	SSIM	PSNR	SSIM	PSNR	SSIM
Airplane	30%	34.72	0.973	34.34	0.967	34.34	0.974	29.48	0.919	**37.73**	**0.991**	36.35	0.981
	50%	29.79	0.919	29.23	0.906	30.23	0.940	26.77	0.849	**32.90**	**0.971**	31.53	0.951
	70%	25.24	0.794	24.68	0.777	26.12	0.858	18.63	0.522	**28.57**	0.924	27.07	0.875
Baboon	30%	28.19	0.928	28.62	0.929	30.15	0.954	26.30	0.892	29.80	0.955	**30.76**	**0.961**
	50%	24.75	0.820	24.84	0.821	26.23	0.875	23.81	0.773	26.23	0.873	**26.89**	**0.889**
	70%	22.00	0.644	21.60	0.629	22.71	0.714	17.00	0.394	**23.60**	**0.731**	23.31	0.730
Barbara	30%	34.57	0.973	37.55	0.983	36.41	0.982	29.72	0.924	37.52	0.980	**38.69**	**0.989**
	50%	29.76	0.919	30.86	0.926	30.43	0.930	27.49	0.858	32.45	**0.956**	**32.50**	0.954
	70%	25.39	0.795	25.40	0.784	25.61	0.806	19.79	0.572	**28.38**	**0.889**	27.36	0.856
Facade	30%	37.58	0.989	**39.20**	**0.992**	36.82	0.989	35.22	0.980	37.71	0.989	38.71	**0.992**
	50%	33.49	0.969	**34.60**	**0.973**	31.59	0.960	30.36	0.946	33.21	0.966	33.35	0.971
	70%	29.77	0.925	**30.42**	**0.931**	27.32	0.890	20.84	0.718	29.07	0.901	28.54	0.909
House	30%	30.08	0.977	30.81	0.959	30.02	0.962	32.26	0.941	30.26	0.982	**38.45**	**0.984**
	50%	28.68	0.938	29.14	0.913	28.73	0.930	30.06	0.886	29.37	0.957	**34.20**	**0.958**
	70%	26.17	0.844	26.14	0.800	26.55	0.859	19.65	0.517	27.94	**0.912**	**29.88**	0.893
Peppers	30%	31.34	0.949	30.86	0.931	30.12	0.938	26.74	0.849	**34.64**	**0.981**	34.30	0.970
	50%	27.66	0.882	26.53	0.839	26.59	0.872	25.74	0.810	**30.79**	**0.956**	29.96	0.930
	70%	23.64	0.733	22.06	0.656	22.79	0.733	18.99	0.515	**27.17**	**0.905**	25.76	0.831
Sailboat	30%	31.54	0.959	31.05	0.948	32.33	0.969	27.53	0.898	**33.69**	**0.981**	33.47	0.976
	50%	27.16	0.889	26.74	0.869	27.87	0.915	24.88	0.808	**29.68**	**0.950**	29.07	0.933
	70%	23.13	0.742	22.75	0.711	23.94	0.795	17.56	0.487	**26.02**	**0.882**	25.10	0.830
Giant	30%	30.01	0.953	31.37	0.960	32.13	0.974	27.07	0.905	32.22	0.978	**33.04**	**0.979**
	50%	25.72	0.874	26.65	0.877	27.51	0.919	24.40	0.813	28.05	**0.937**	**28.34**	0.932
	70%	22.03	0.717	22.46	0.713	23.44	0.797	17.65	0.502	**24.50**	**0.844**	24.25	0.818
Tiger	30%	33.16	0.976	37.58	0.988	37.55	0.991	28.39	0.918	36.97	0.990	**39.29**	**0.994**
	50%	27.89	0.914	30.12	0.936	30.65	0.955	25.19	0.823	31.13	0.958	**32.39**	**0.969**
	70%	23.34	0.763	24.33	0.779	25.13	0.843	18.01	0.516	**26.55**	0.873	26.48	**0.877**

**Table 2:** The PSNR and SSIM Comparison of different methods on the Kodak PhotoCD dataset, the incomplete images tested contain 30 percent missing entries (The best results are highlighted).

Test Images	FaLRTC [4]		LRTC-TNN1 [5]		LRTC-TNN2 [5]		TCTF [6]		LRTV-TTr1 [7]		SLRTC	
	PSNR	SSIM	PSNR	SSIM	PSNR	SSIM	PSNR	SSIM	PSNR	SSIM	PSNR	SSIM
kodim01	33.57	0.989	39.68	0.990	39.06	0.991	29.03	0.927	35.98	**0.997**	**41.01**	0.994
kodim02	38.42	0.992	41.80	0.987	41.31	0.989	33.28	0.942	38.58	**0.996**	**44.39**	0.994
kodim03	38.33	0.994	40.75	0.987	42.68	0.995	31.87	0.924	37.82	**0.997**	**44.64**	0.996
kodim04	38.16	0.993	40.92	0.986	34.28	0.984	31.76	0.921	37.89	**0.997**	**43.58**	0.994
kodim05	31.12	0.989	35.82	0.983	36.67	0.990	24.80	0.858	33.99	**0.996**	**38.98**	0.994
kodim06	34.57	0.989	39.60	0.989	41.20	0.995	29.76	0.925	36.02	**0.996**	**42.20**	**0.996**
kodim07	37.71	0.996	41.08	0.990	42.34	0.995	30.13	0.910	37.64	**0.998**	**44.41**	0.996
kodim08	31.63	0.991	37.02	0.987	34.59	0.987	25.45	0.897	34.95	**0.997**	**37.82**	0.993
kodim09	38.49	0.995	42.38	0.990	34.33	0.987	32.38	0.943	38.11	**0.998**	**43.85**	0.995
kodim10	37.45	0.994	41.53	0.989	34.00	0.987	31.90	0.935	37.95	**0.998**	**43.43**	0.995
kodim11	34.83	0.991	39.48	0.988	40.39	0.993	30.37	0.929	36.40	**0.996**	**42.18**	0.995
kodim12	39.11	0.994	43.00	0.990	42.59	0.994	34.10	0.946	38.71	**0.997**	**44.80**	0.995
kodim13	29.23	0.981	34.58	0.982	36.30	0.989	25.09	0.885	32.61	**0.993**	**37.07**	0.991
kodim14	33.18	0.989	36.35	0.981	37.92	0.990	27.51	0.890	34.70	**0.995**	**39.83**	0.993
kodim15	37.09	0.992	40.39	0.985	38.97	0.987	31.00	0.923	38.01	**0.997**	**42.03**	0.993
kodim16	38.77	0.993	43.40	0.992	45.51	0.996	33.28	0.944	37.72	**0.997**	**46.23**	**0.997**
kodim17	36.72	0.994	40.56	0.989	41.69	0.994	31.14	0.925	41.31	**0.998**	**42.79**	0.995
kodim18	32.57	0.987	36.62	0.980	36.91	0.987	27.89	0.888	36.50	**0.996**	**38.60**	0.991
kodim19	36.34	0.992	40.81	0.988	39.06	0.990	30.86	0.927	40.60	**0.997**	**41.81**	0.994
kodim20	36.56	0.994	40.02	0.988	40.73	0.992	31.08	0.939	37.77	**0.998**	**42.12**	0.994
kodim21	34.77	0.993	39.06	0.987	40.73	0.993	29.06	0.924	35.98	**0.997**	**41.58**	0.994
kodim22	35.79	0.990	38.93	0.983	38.30	0.986	30.78	0.915	36.88	**0.995**	**40.16**	0.990
kodim23	37.39	0.996	36.61	0.990	37.59	0.989	31.66	0.921	37.13	**0.998**	**44.17**	0.994
kodim24	31.51	0.987	34.76	0.978	35.68	0.986	27.17	0.896	35.28	**0.996**	**36.77**	0.990

**Table 3:** The PSNR and SSIM Comparison of different methods on the Kodak PhotoCD dataset, the incomplete images tested contain 50 percent missing entries (The best results are highlighted).

Test Images	FaLRTC [4]		LRTC-TNN1 [5]		LRTC-TNN2 [5]		TCTF [6]		LRTV-TTr1 [7]		SLRTC	
	PSNR	SSIM	PSNR	SSIM	PSNR	SSIM	PSNR	SSIM	PSNR	SSIM	PSNR	SSIM
kodim01	29.05	0.959	32.58	0.948	32.31	0.958	26.67	0.851	31.82	**0.984**	**34.01**	0.970
kodim02	33.84	0.971	35.22	0.944	35.43	0.960	31.59	0.890	35.36	**0.985**	**37.67**	0.972
kodim03	33.77	0.975	34.64	0.944	37.05	0.977	29.50	0.869	35.10	**0.989**	**39.05**	0.983
kodim04	33.30	0.971	34.34	0.939	32.33	0.957	29.36	0.857	35.31	**0.988**	**37.05**	0.972
kodim05	26.01	0.949	28.34	0.905	29.98	0.950	22.45	0.742	29.08	**0.981**	**31.93**	0.967
kodim06	29.81	0.958	32.80	0.946	34.92	0.976	27.20	0.849	31.92	**0.981**	**35.67**	0.979
kodim07	32.52	0.981	34.00	0.952	35.59	0.975	27.78	0.847	34.19	**0.992**	**37.65**	0.984
kodim08	26.74	0.963	29.74	0.933	28.07	0.939	23.06	0.806	30.00	**0.986**	**30.84**	0.965
kodim09	33.55	0.981	35.66	0.958	32.21	0.966	29.95	0.892	35.33	**0.992**	**37.11**	0.978
kodim10	32.66	0.976	34.80	0.950	31.90	0.963	29.48	0.877	34.70	**0.992**	**36.89**	0.978
kodim11	30.31	0.965	32.65	0.940	33.94	0.967	27.90	0.858	32.56	**0.984**	**35.38**	0.974
kodim12	34.21	0.975	36.19	0.956	36.60	0.975	31.62	0.896	35.23	**0.987**	**38.67**	0.981
kodim13	24.86	0.927	27.91	0.908	30.02	0.949	22.47	0.763	27.71	**0.968**	**30.62**	0.957
kodim14	28.53	0.956	29.60	0.910	31.71	0.956	25.11	0.793	30.47	**0.979**	**33.35**	0.967
kodim15	32.43	0.972	33.83	0.936	33.08	0.953	28.65	0.864	34.65	**0.989**	**35.96**	0.970
kodim16	33.97	0.973	36.84	0.964	39.39	0.983	30.89	0.887	34.89	**0.988**	**39.95**	0.985
kodim17	32.23	0.975	34.58	0.953	36.06	0.975	28.69	0.858	36.43	**0.993**	**37.34**	0.981
kodim18	28.04	0.951	29.99	0.908	30.89	0.944	25.50	0.788	31.29	**0.981**	**32.47**	0.961
kodim19	31.68	0.971	34.17	0.949	32.50	0.960	28.50	0.861	**35.21**	**0.988**	35.11	0.972
kodim20	31.51	0.975	33.64	0.950	34.72	0.971	28.64	0.884	33.95	**0.991**	**36.13**	0.977
kodim21	29.71	0.971	32.41	0.943	34.52	0.972	26.70	0.856	31.95	**0.987**	**35.34**	0.976
kodim22	31.31	0.963	32.87	0.931	32.91	0.948	28.38	0.839	33.10	**0.981**	**34.56**	0.963
kodim23	33.35	0.985	33.47	0.960	33.72	0.964	29.21	0.873	34.46	**0.992**	**37.96**	0.978
kodim24	27.10	0.951	29.10	0.908	30.47	0.945	24.75	0.799	30.31	**0.984**	**31.45**	0.959

**Table 4:** The PSNR and SSIM Comparison of different methods on the Kodak PhotoCD dataset, the incomplete images tested contain 70 percent missing entries (The best results are high-lighted).

Test Images	FaLRTC [4]		LRTC-TNN1 [5]		LRTC-TNN2 [5]		TCTF [6]		LRTV-TTr1 [7]		SLRTC	
	PSNR	SSIM	PSNR	SSIM	PSNR	SSIM	PSNR	SSIM	PSNR	SSIM	PSNR	SSIM
kodim01	25.42	0.876	26.84	0.819	26.98	0.857	22.92	0.699	27.20	**0.932**	**28.29**	0.886
kodim02	30.24	0.919	30.46	0.848	31.00	0.894	28.80	0.796	31.67	**0.950**	**32.60**	0.913
kodim03	29.69	0.924	29.48	0.841	31.63	0.922	27.48	0.772	31.11	**0.963**	**33.59**	0.942
kodim04	29.05	0.910	29.12	0.822	29.37	0.884	26.11	0.738	31.18	**0.956**	**31.66**	0.911
kodim05	21.80	0.827	22.67	0.711	24.59	0.832	19.41	0.538	24.48	**0.925**	**26.14**	0.874
kodim06	26.09	0.871	27.48	0.828	29.78	0.921	23.19	0.694	27.74	**0.927**	**30.24**	0.924
kodim07	27.85	0.927	28.35	0.849	29.93	0.913	24.71	0.726	30.07	**0.971**	**31.86**	0.939
kodim08	22.47	0.881	23.88	0.791	22.93	0.815	20.18	0.664	**25.12**	**0.946**	24.89	0.867
kodim09	28.99	0.936	29.96	0.874	28.71	0.905	25.61	0.786	31.08	**0.971**	**31.34**	0.927
kodim10	28.65	0.923	29.42	0.850	28.82	0.902	25.59	0.759	30.94	**0.971**	**31.52**	0.928
kodim11	26.56	0.897	27.38	0.822	28.71	0.893	23.70	0.714	28.43	**0.942**	**29.74**	0.906
kodim12	30.14	0.926	30.89	0.872	31.34	0.925	27.37	0.785	31.66	**0.957**	**33.03**	0.935
kodim13	21.49	0.791	22.78	0.722	25.04	0.837	18.82	0.550	23.54	**0.886**	**25.45**	0.852
kodim14	24.57	0.856	24.59	0.740	26.69	0.859	22.42	0.623	26.34	**0.924**	**28.02**	0.883
kodim15	28.28	0.917	28.58	0.823	28.15	0.865	26.14	0.761	30.47	**0.963**	**30.58**	0.905
kodim16	30.00	0.913	31.25	0.875	34.03	0.942	26.38	0.743	31.09	**0.950**	**34.35**	0.945
kodim17	28.11	0.914	29.24	0.853	30.85	0.915	24.33	0.718	31.67	**0.972**	**32.17**	0.936
kodim18	24.42	0.850	25.06	0.743	26.26	0.834	21.35	0.598	27.02	**0.932**	**27.63**	0.873
kodim19	27.50	0.910	28.61	0.841	27.26	0.877	24.81	0.736	**30.32**	**0.958**	29.18	0.904
kodim20	27.40	0.925	28.34	0.857	29.92	0.919	24.76	0.777	29.85	**0.970**	**31.26**	0.932
kodim21	25.64	0.905	26.89	0.831	29.23	0.914	22.64	0.715	27.68	**0.952**	**29.83**	0.920
kodim22	27.55	0.887	27.99	0.803	28.36	0.853	25.09	0.694	29.23	**0.935**	**29.72**	0.883
kodim23	29.09	0.951	28.94	0.880	29.14	0.898	26.94	0.817	30.89	**0.976**	**32.33**	0.932
kodim24	23.61	0.857	24.47	0.752	26.00	0.840	21.21	0.607	26.23	**0.941**	**26.84**	0.869

## Data Availability

Data available on request from the authors.
